# Supplementary material for: Neural stem cell-derived extracellular vesicles drive early neuroprotective and anti-apoptotic responses in spinal cord injury organotypic slices
Source: Front Cell Neurosci. 2026 Jun 3;20:1835240. doi: 10.3389/fncel.2026.1835240 (PMC13272055; doi:10.3389/fncel.2026.1835240)

Original images of the blots for Figure 1

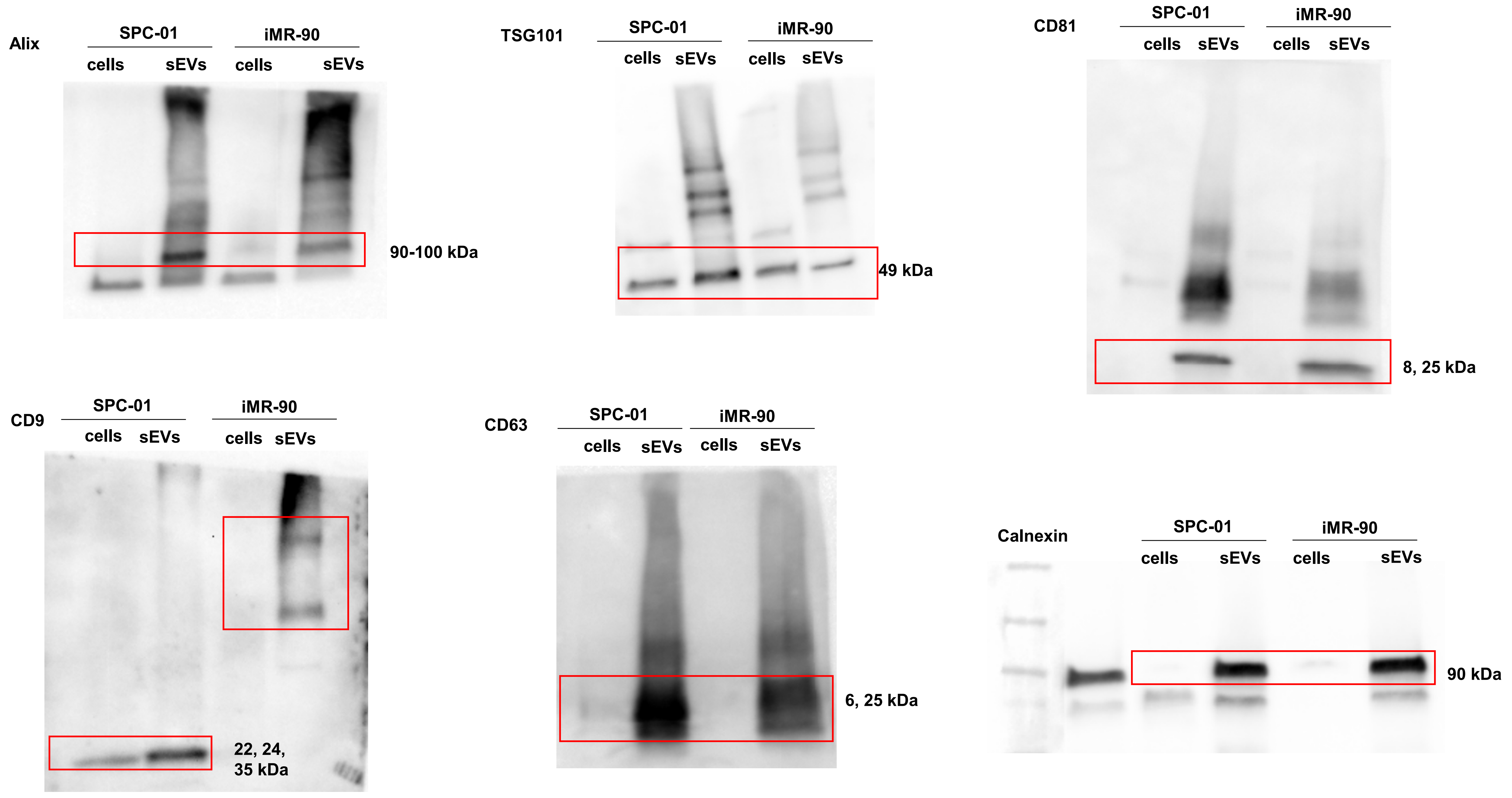

Original images of the blots for Figure 3A

(red frame labels the representative image included to the Figure; green frame labels replicates included to quantification)

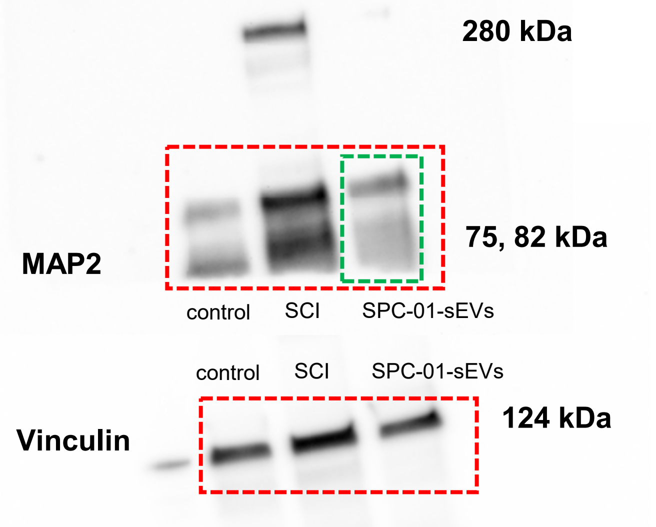

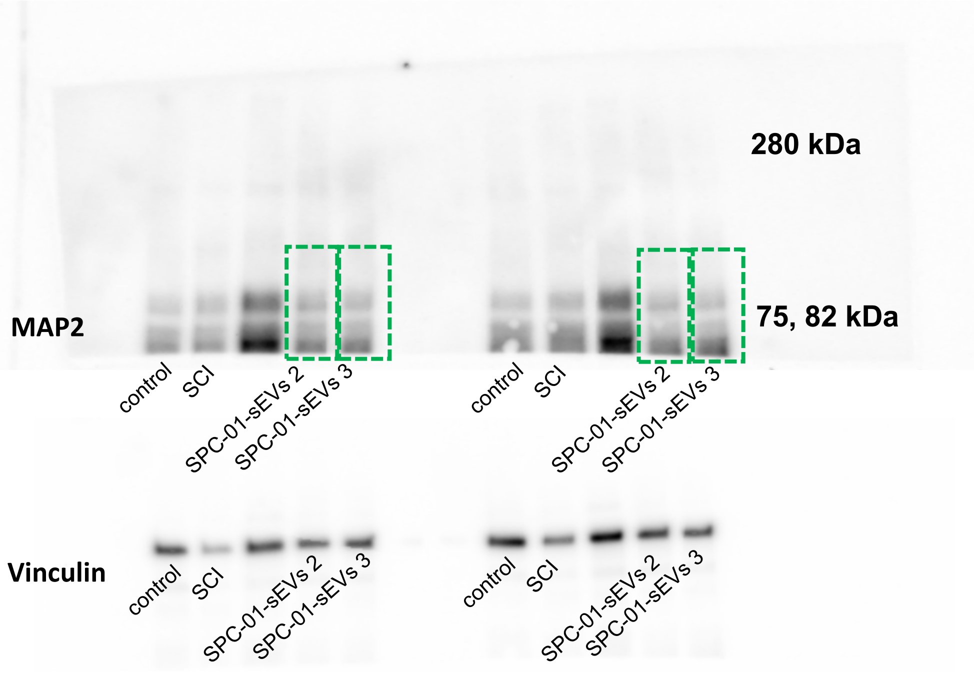

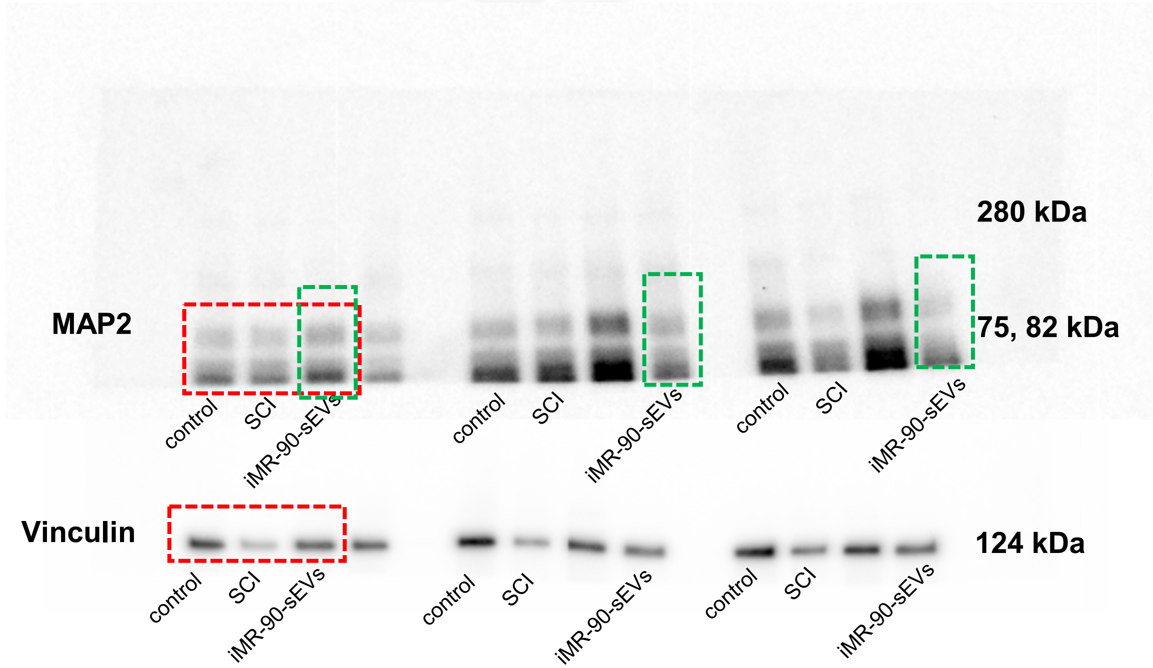

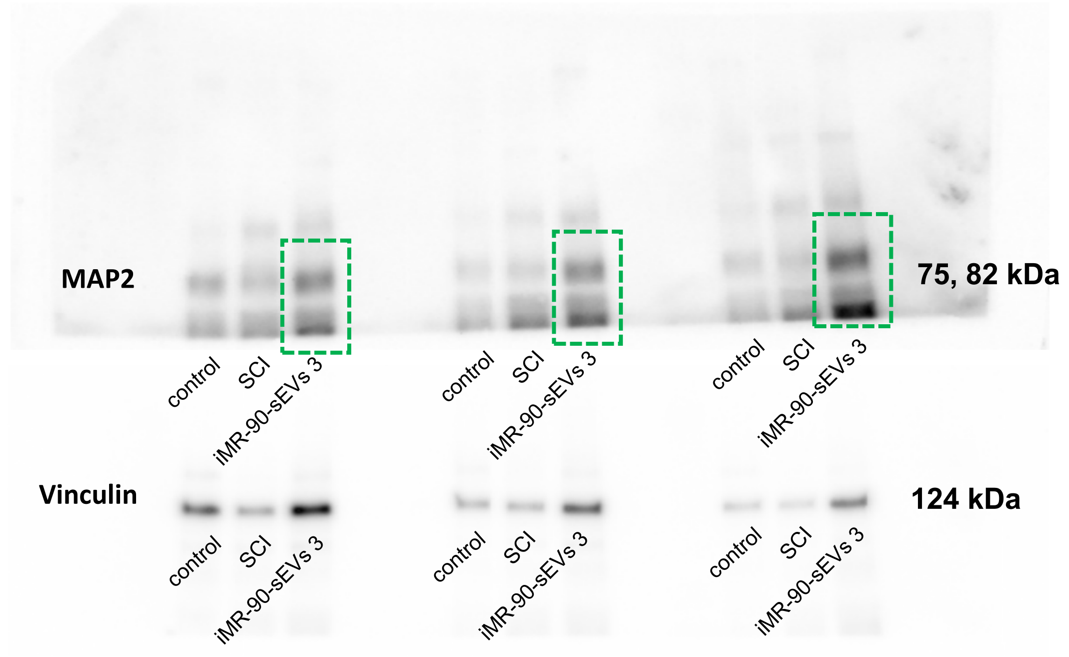

Original images of the blots for Figure 3B

(red frame labels the representative image included to the Figure; green frame labels replicates included to quantification)

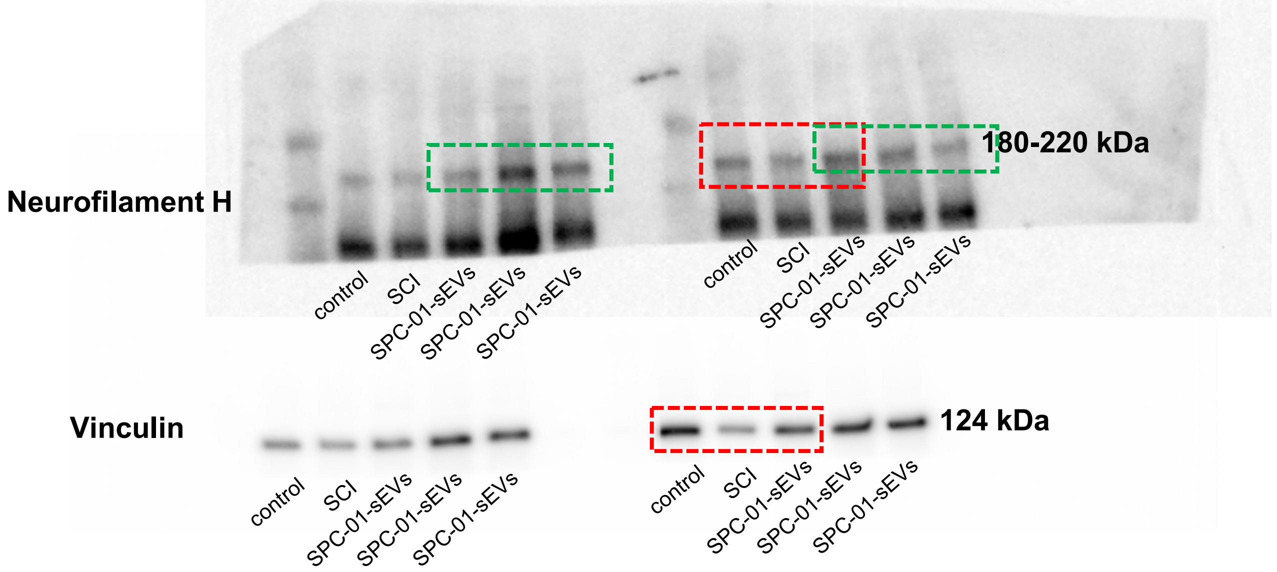

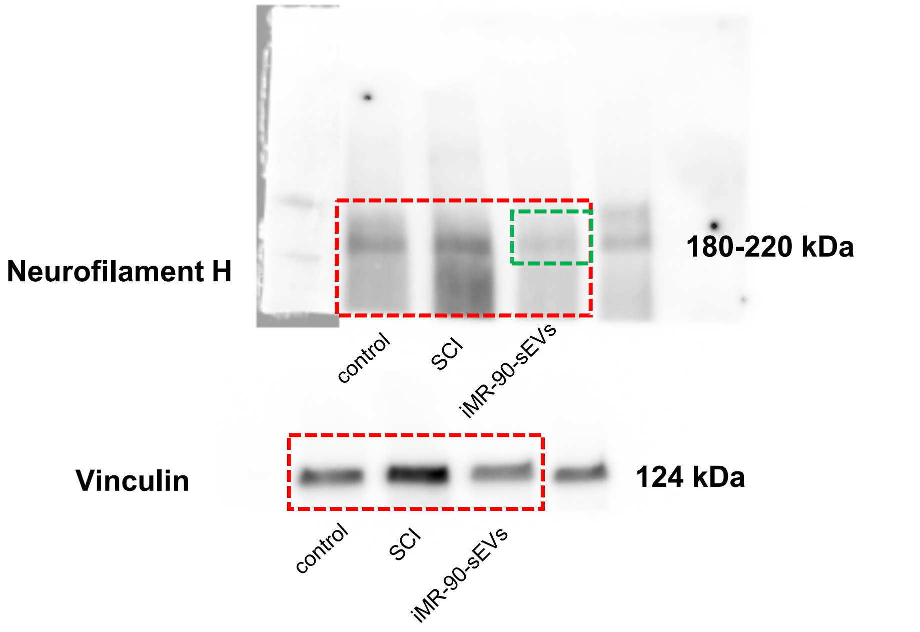

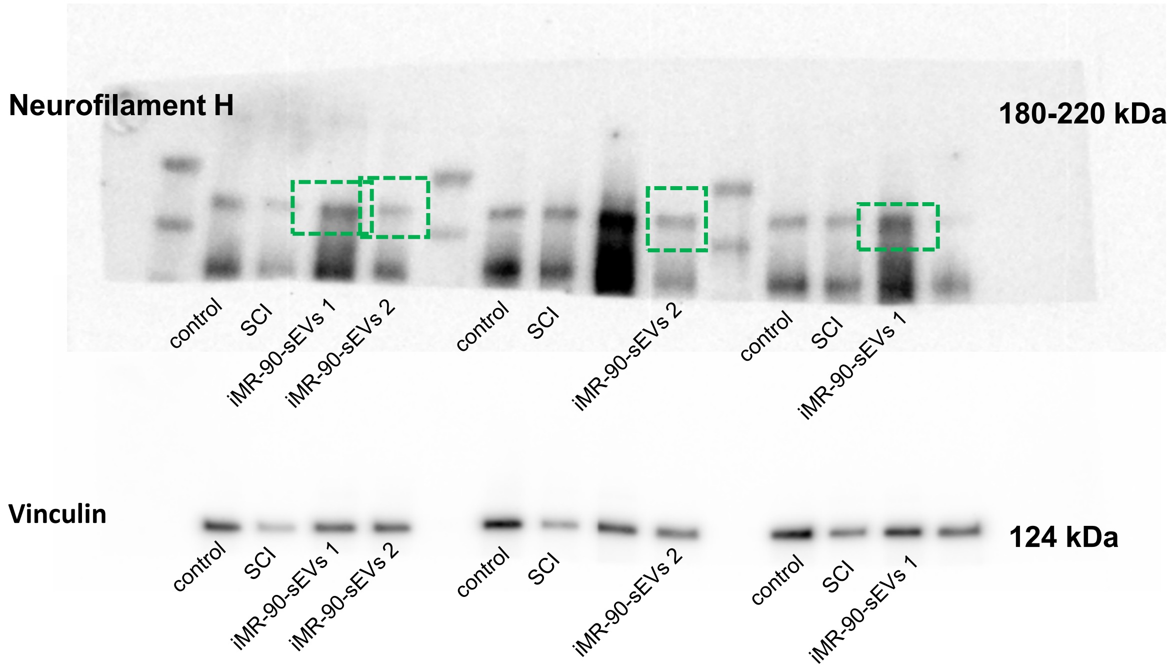

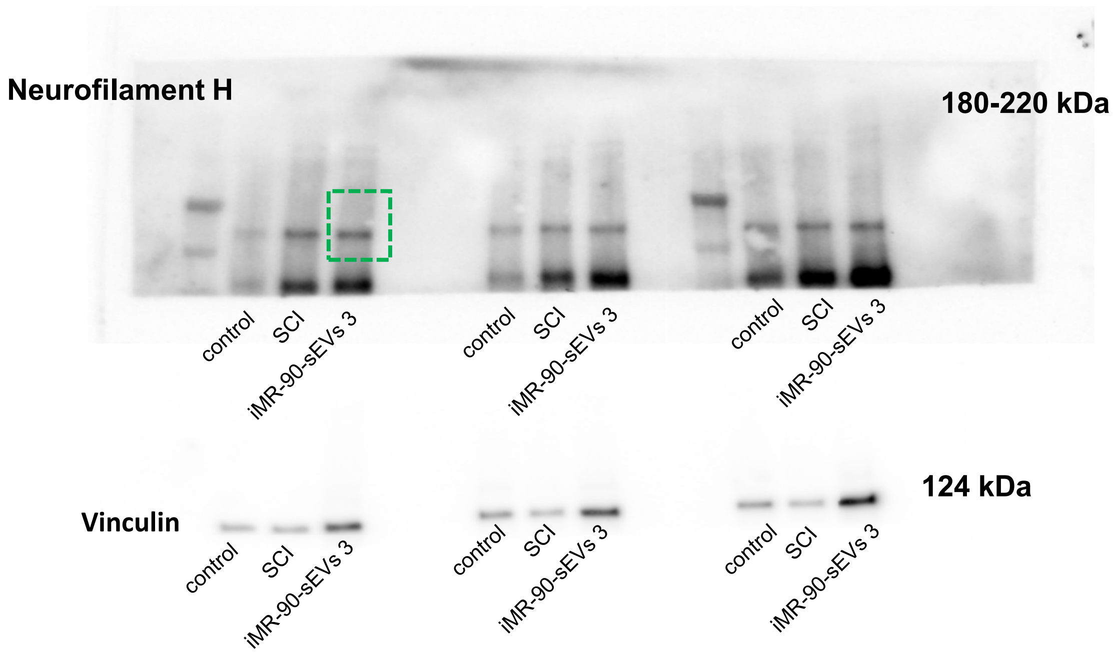

Original images of the blots for Figure 3C

(red frame labels the representative image included to the Figure; green frame labels replicates included to quantification)

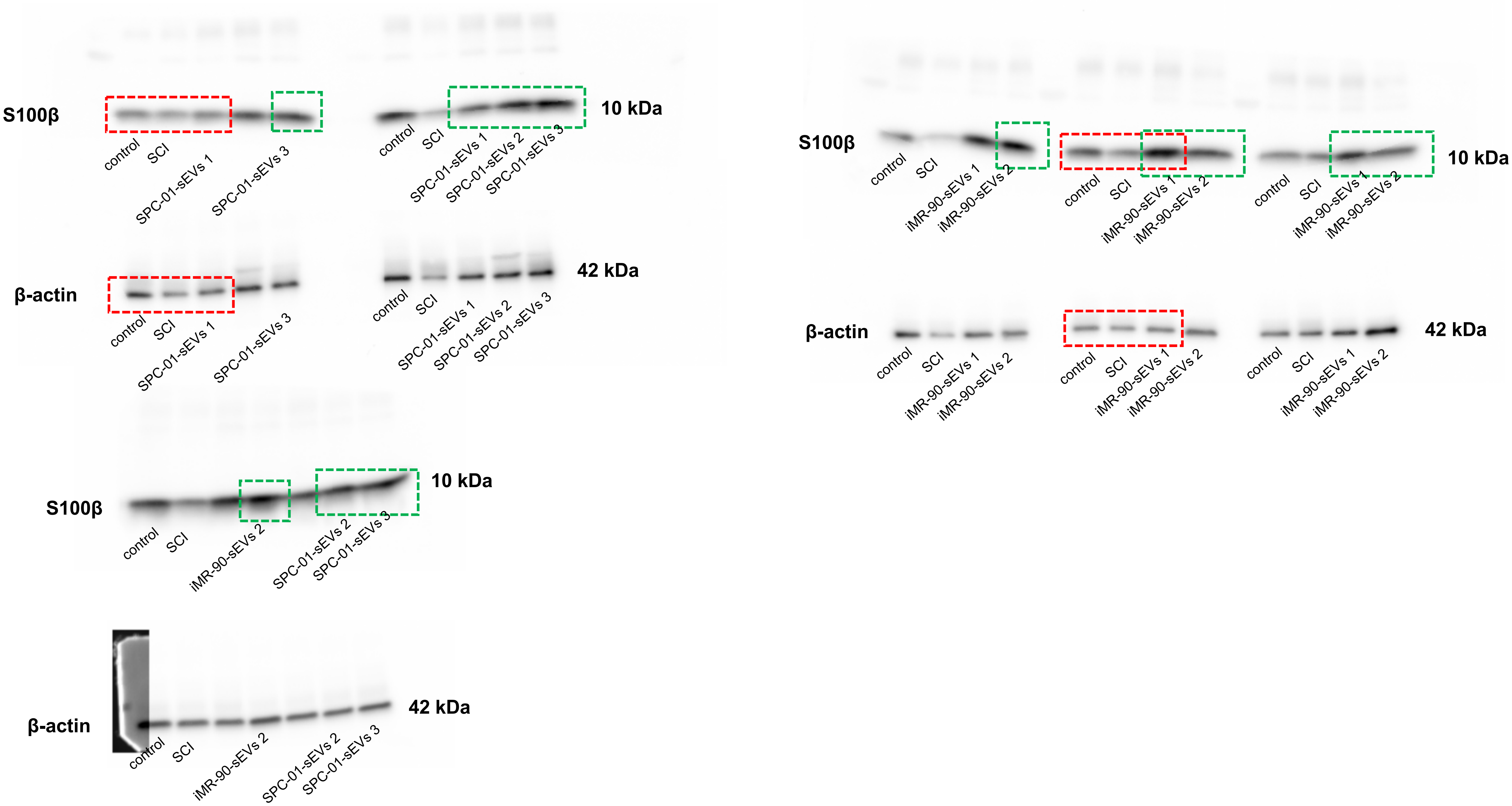

Original images of the blots for Figures 3D

(red frame labels the representative image included to the Figure; green frame labels replicates included to quantification)

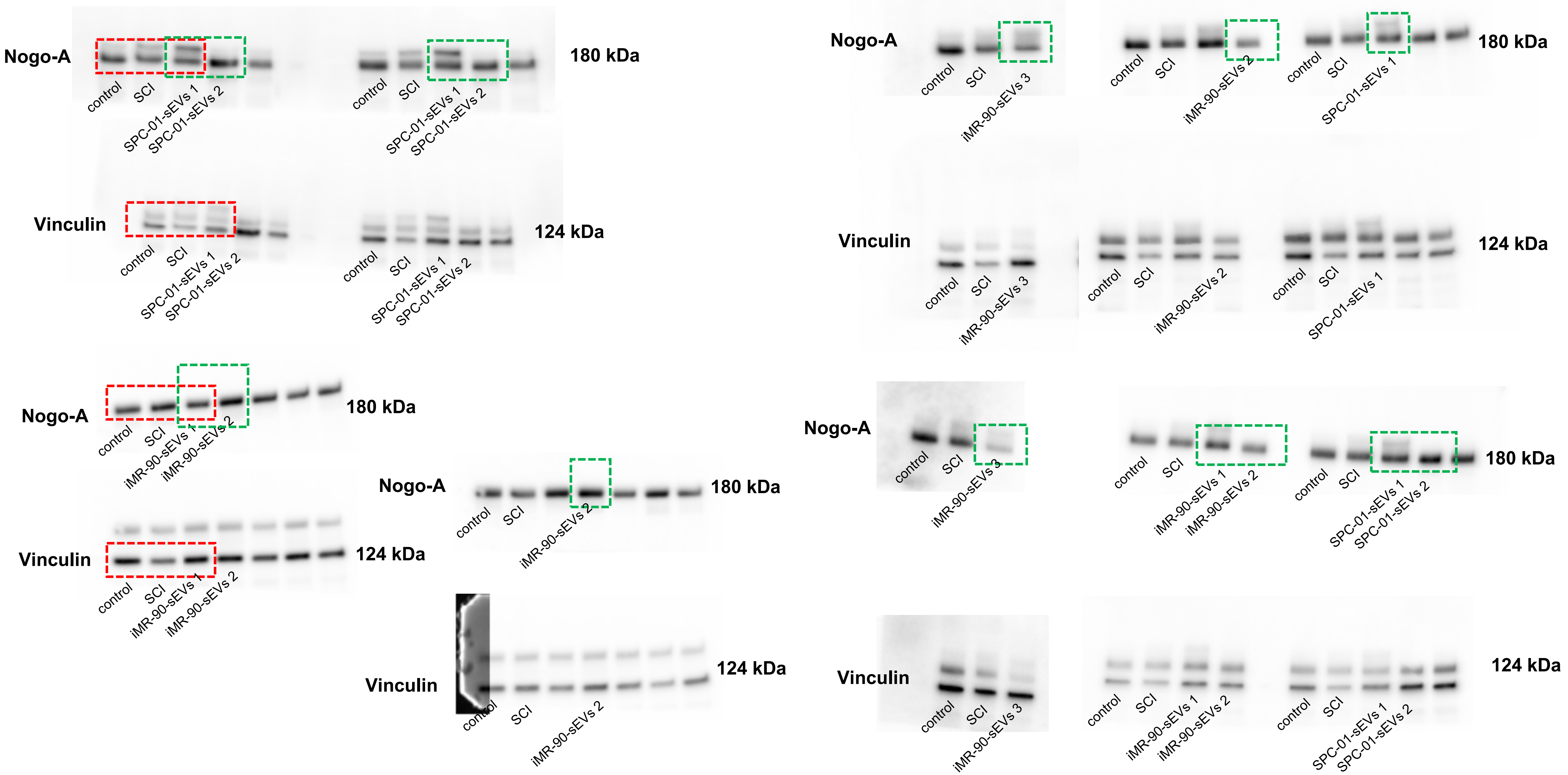

Original images of the blots for Figure 3E

(red frame labels the representative image included to the Figure; green frame labels replicates included to quantification)

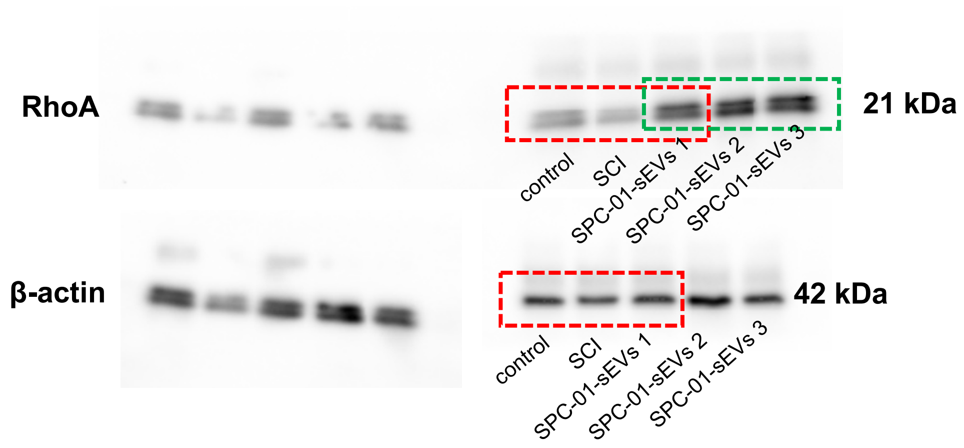

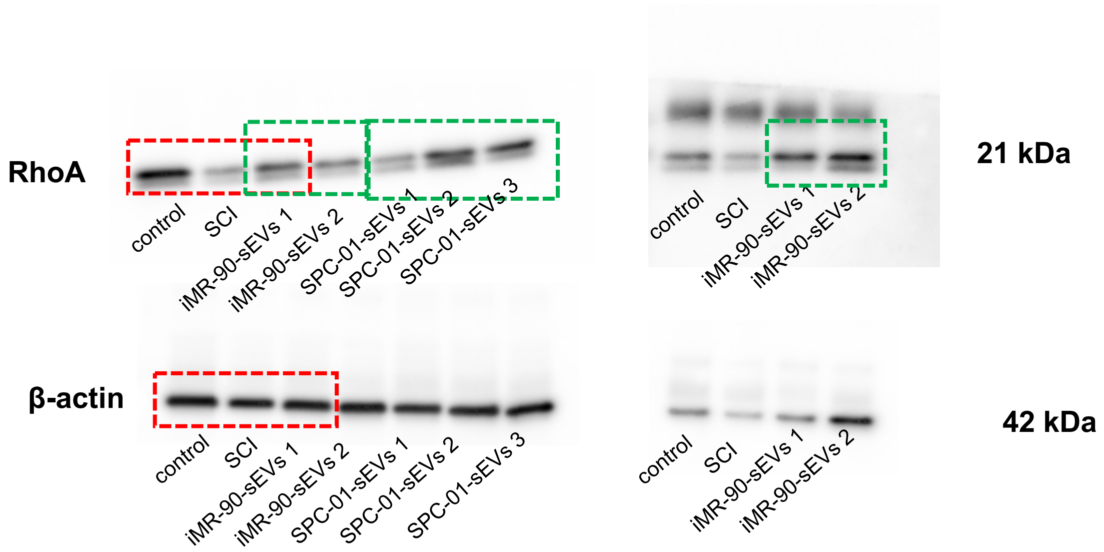

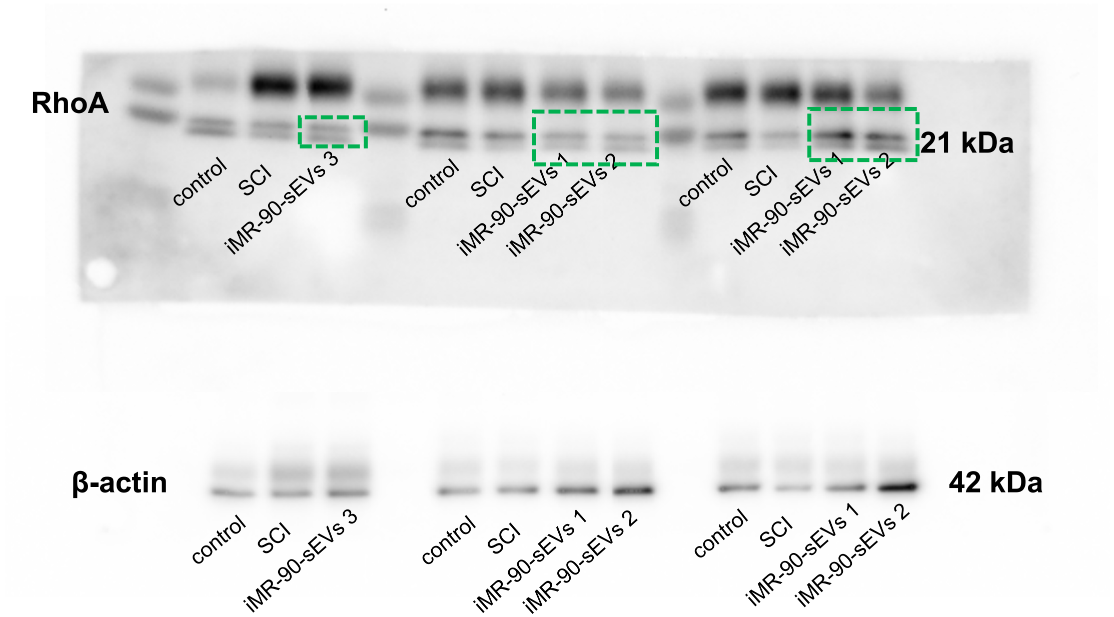

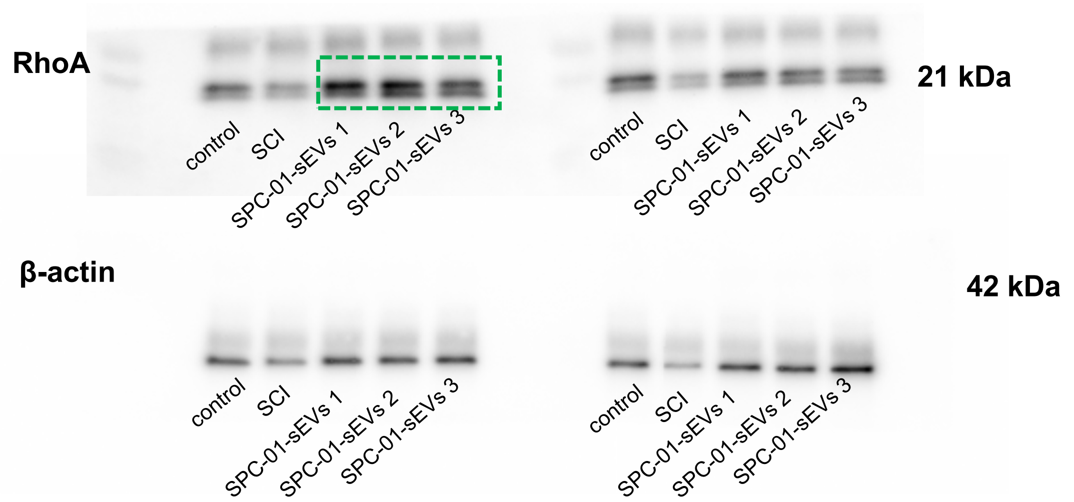

Original images of the blots for Figure 4A

(red frame labels the representative image included to the Figure; green frame labels replicates included to quantification)

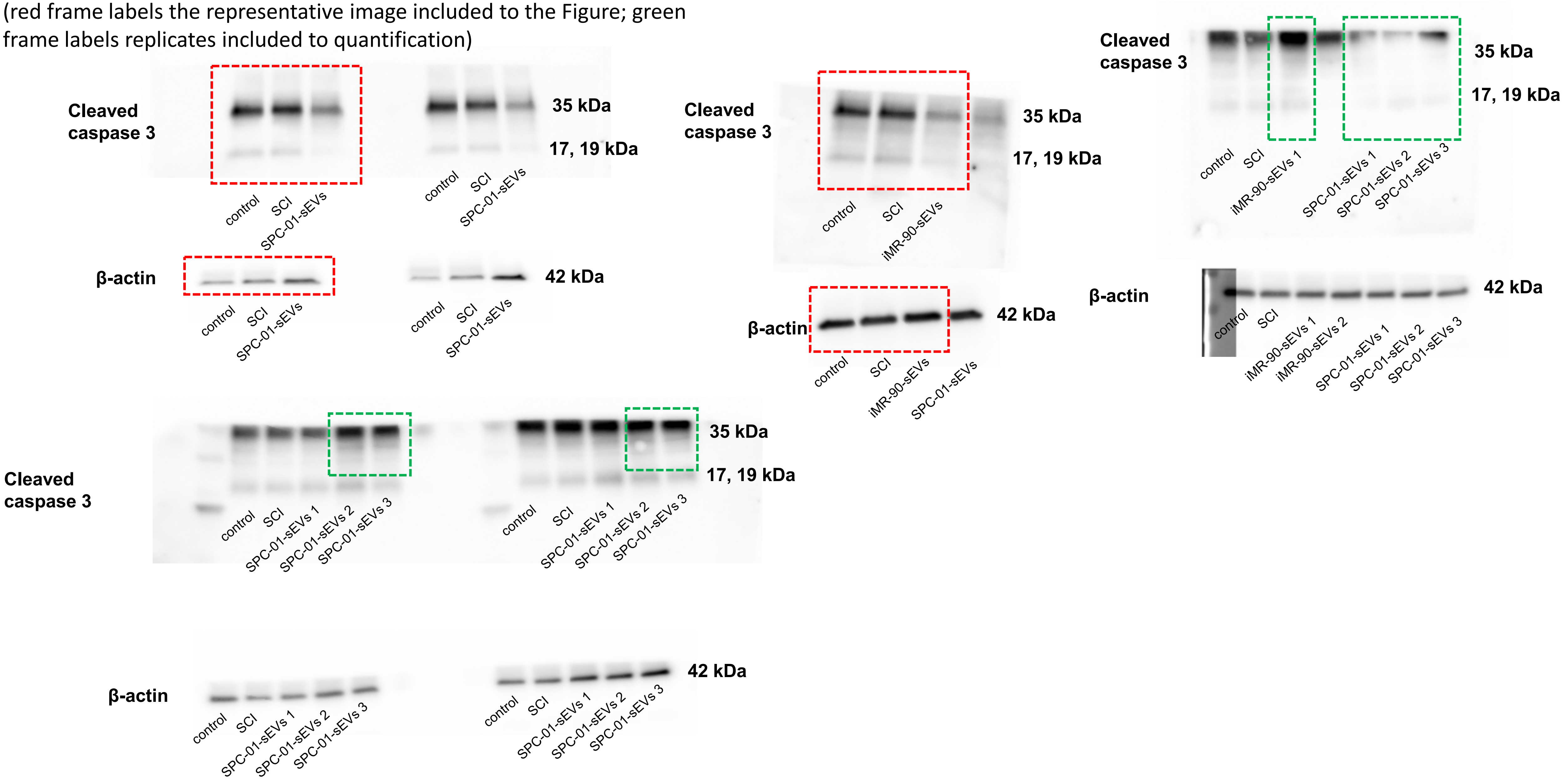

Original images of the blots for Figure 4A
(red frame labels the representative image included to the Figure; green frame labels replicates included to quantification)

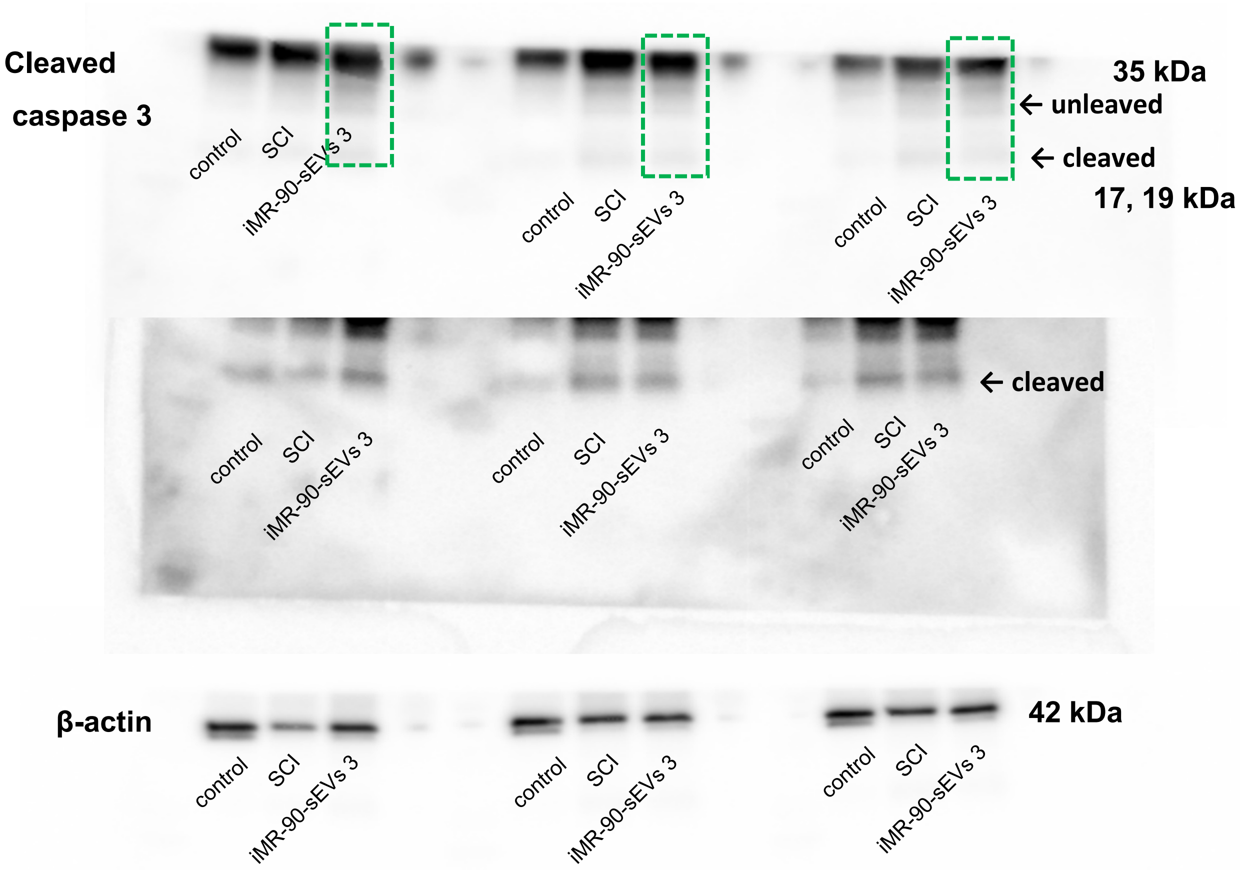

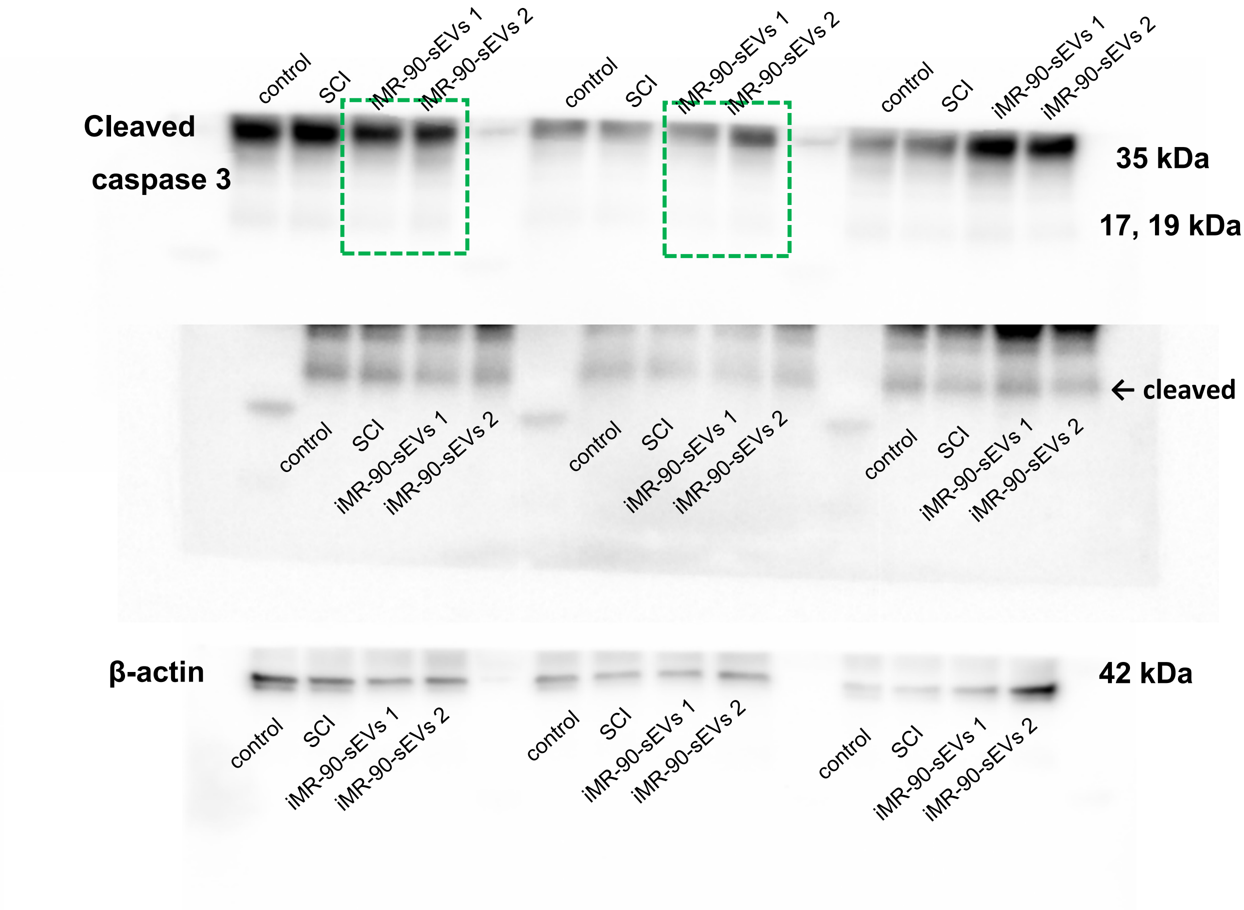

Original images of the blots for Figure 4B

(red frame labels the representative image included to the Figure;
green frame labels replicates included to quantification)

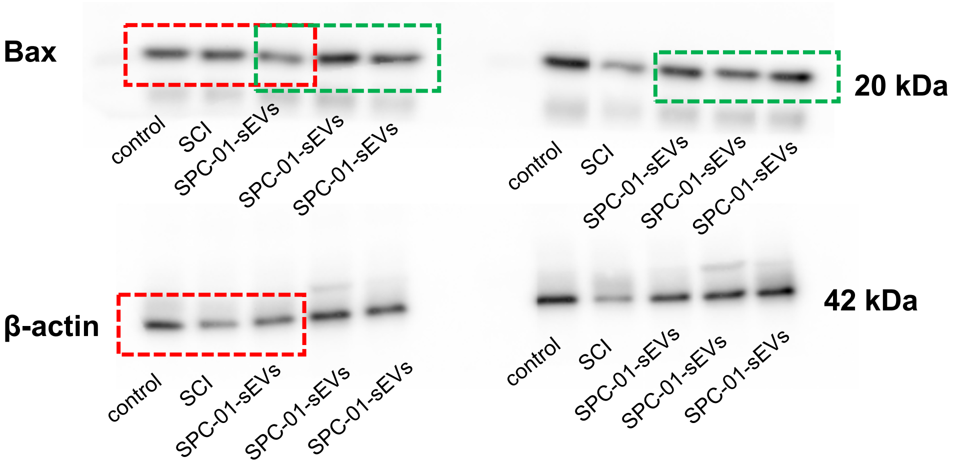

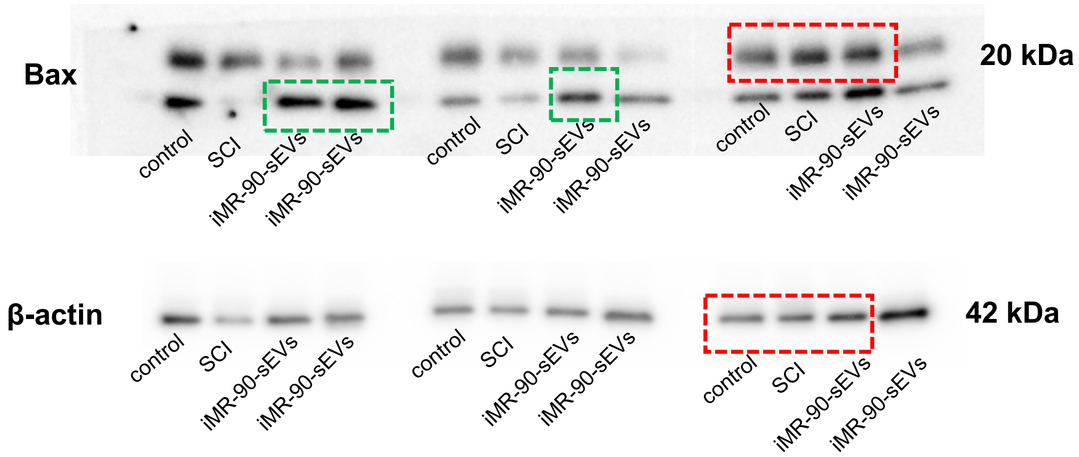

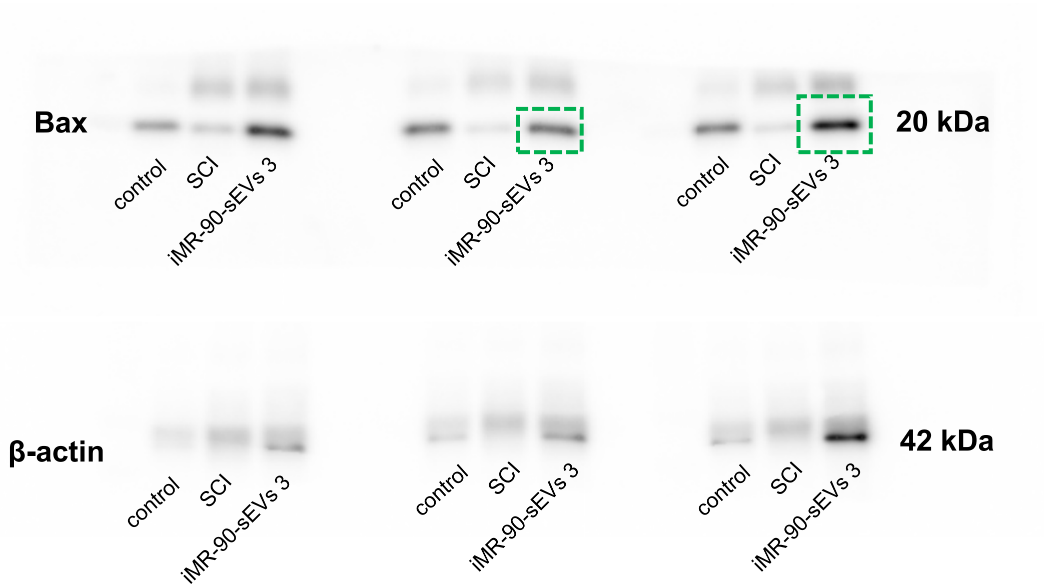

Original images of the blots for Figure 4C

(red frame labels the representative image included to the Figure;
green frame labels replicates included to quantification)

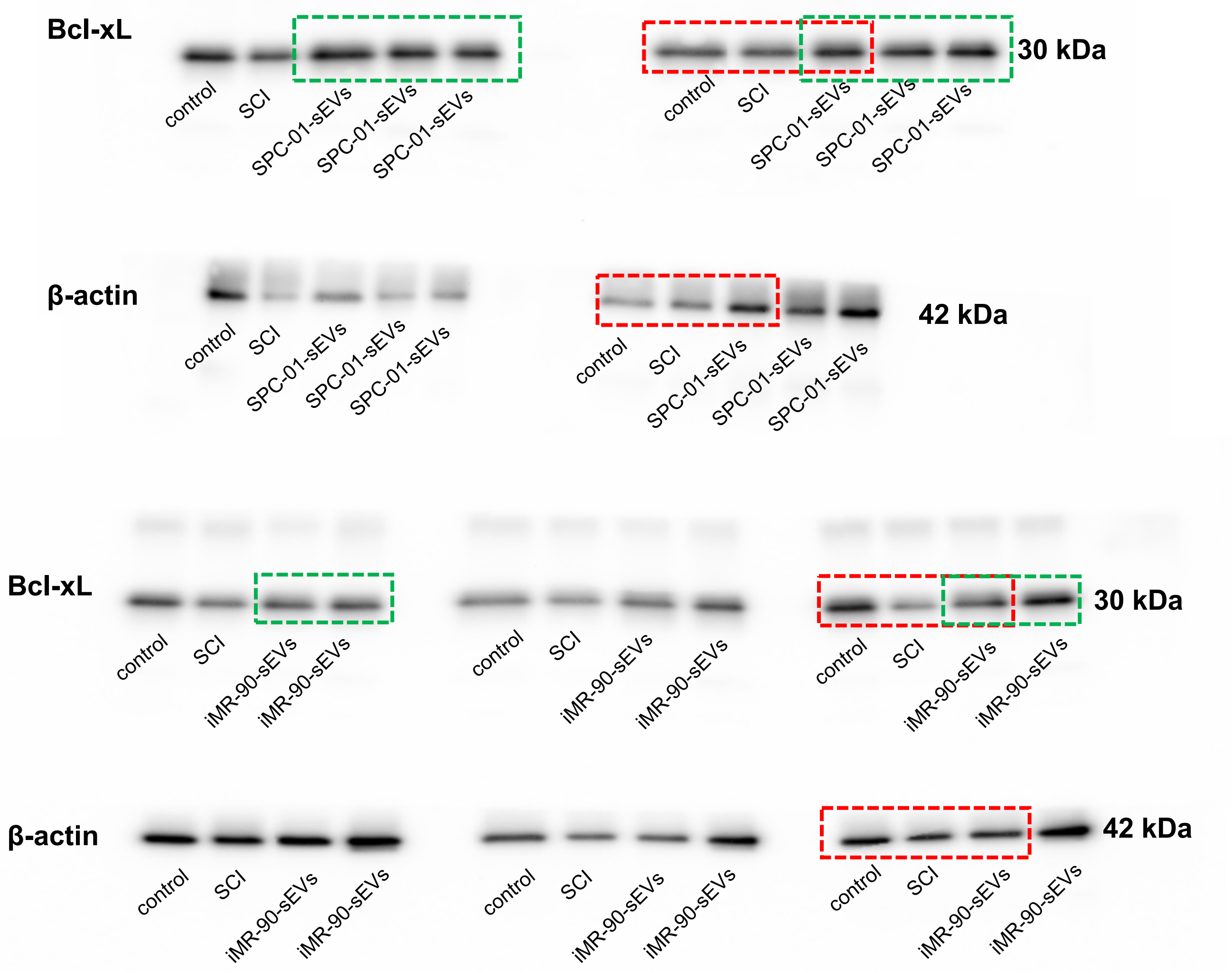

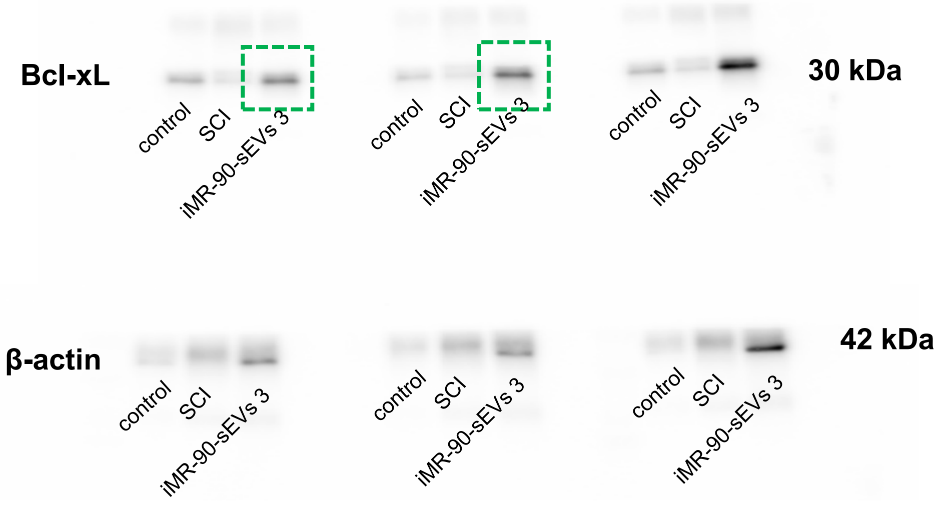

Original images of the blots for Figures 4D

(red frame labels the representative image included to the Figure;
green frame labels replicates included to quantification)

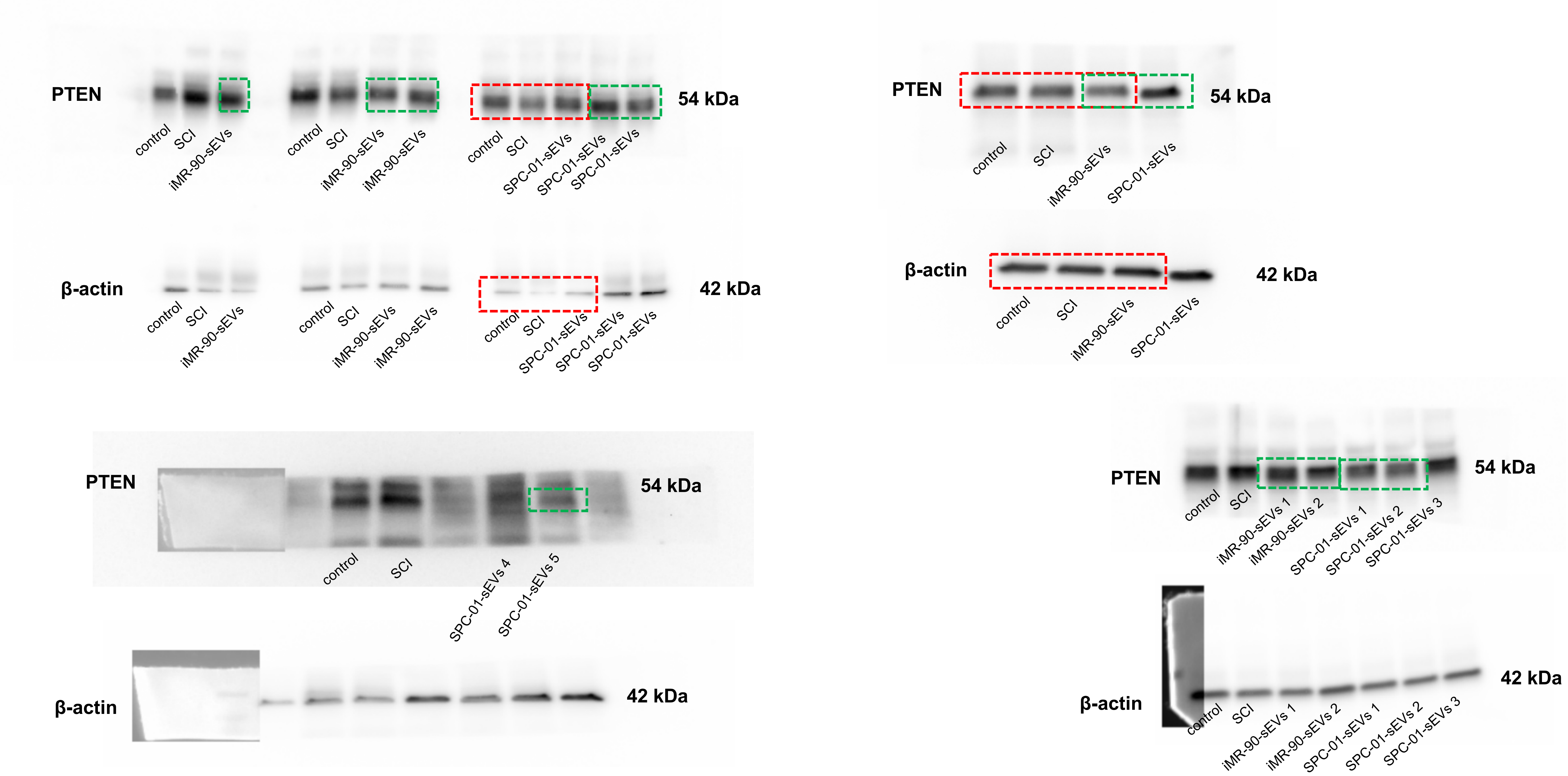

Original images of the blots for Figure 4E (red frame labels the representative image included to the Figure; green frame labels replicates included to quantification)

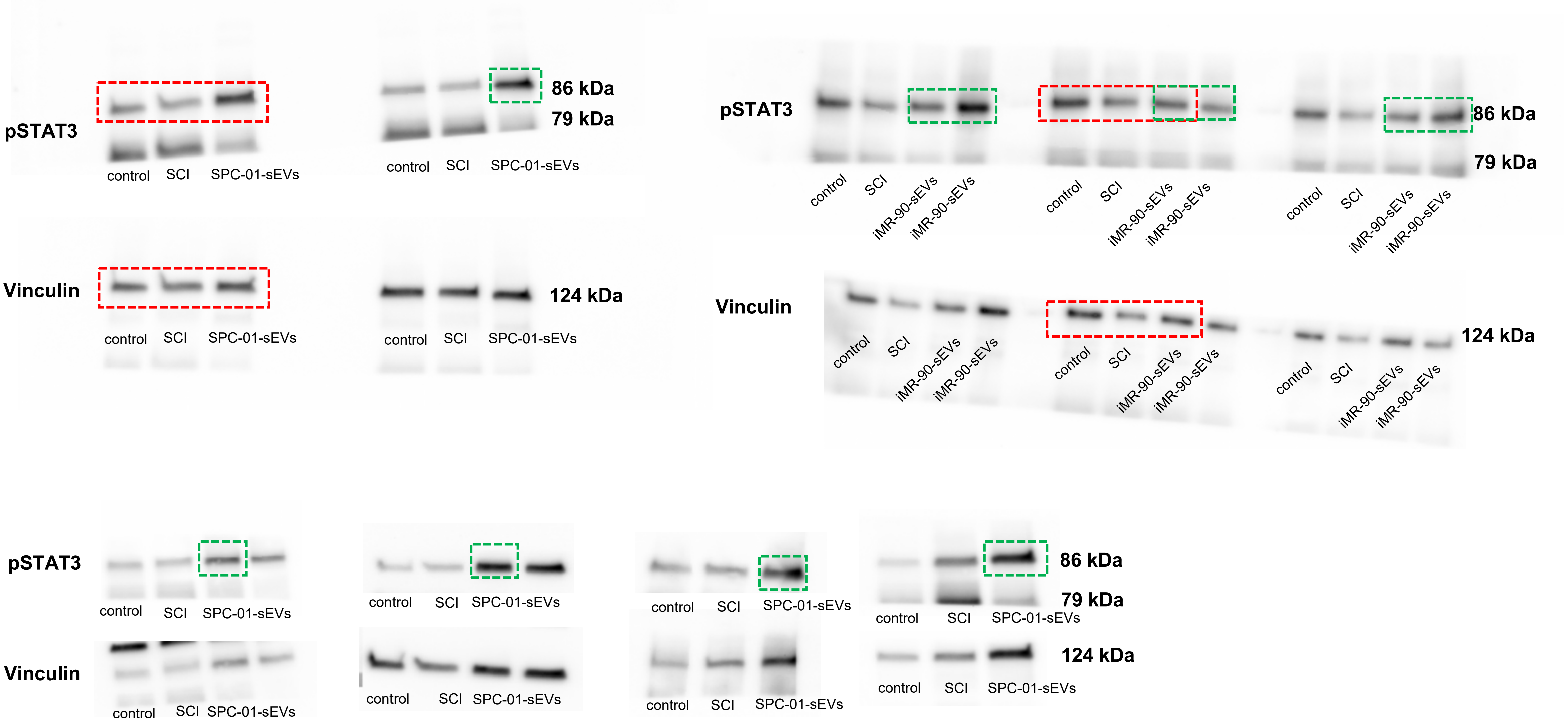

Supplement: Supplementary file 1 [file Data_Sheet_1.pdf]
