# Supplementary material for: Neural stem cell-derived extracellular vesicles drive early neuroprotective and anti-apoptotic responses in spinal cord injury organotypic slices
Source: Front Cell Neurosci. 2026 Jun 3;20:1835240. doi: 10.3389/fncel.2026.1835240 (PMC13272055; doi:10.3389/fncel.2026.1835240)
Supplement: Supplementary file 2 [file Table_1.docx]

***Supplementary table 2:*** ***List of secondary antibodies used for Western Blot and Immunohistochemistry (IHC).***

| Antibody | Dilution | Catalog number |
| --- | --- | --- |
| Goat Anti-Rabbit | 1:10 000 | 131599 (Sigma Aldrich) |
| Goat Anti-Mouse | 1:10 000 | 131224 (Sigma Aldrich) |
| Goat anti-Rabbit IgG (H+L) Alexa Fluor™ 594 | 1:400 | A11037 (Thermo Fisher) |
| Goat anti-Mouse IgG (H+L) Alexa Fluor™ 594 | 1:400 | A11032 (Thermo Fisher) |
| Goat anti-Mouse IgG (H+L) Alexa Fluor™ 488 | 1:400 | A11029 (Thermo Fisher) |
| Goat anti-Rabbit IgG (H+L) Alexa Fluor™ 488 | 1:400 | A11034 (Thermo Fisher) |
