# Supplementary material for: Neural stem cell-derived extracellular vesicles drive early neuroprotective and anti-apoptotic responses in spinal cord injury organotypic slices
Source: Front Cell Neurosci. 2026 Jun 3;20:1835240. doi: 10.3389/fncel.2026.1835240 (PMC13272055; doi:10.3389/fncel.2026.1835240)
Supplement: Supplementary file 3 [file Table_2.docx]

***Supplementary table 1:*** ***List of primary antibodies used for Western Blot and Immunohistochemistry (IHC).*** *All antibodies are from Cell Signaling Technology, Danvers, MA, USA, unless stated otherwise.*

| Antibody | IHC | Western blot | Molecular weight (kDa) | Catalog number |
| --- | --- | --- | --- | --- |
| MAP2 | 1:50 | 1:1000, BSA | 75, 82, 280 | 4542 |
| Neurofilament-H | 1:400 | 1:1000, milk | 180-220 | 2836 |
| Nogo-A | - | 1:1000, BSA | 180 | 13401 |
| Vinculin | - | 1:1000, BSA | 124 | 13901 |
| Alix | - | 1:1000, milk | 90-100 | 92880 |
| Calnexin | - | 1:1000, BSA | 90 | 10427-2-AP  (proteintech) |
| pSTA3 | - | 1:1000, BSA | 79, 86 | 9145 |
| STAT3 | - | 1:1000, milk | 79, 86 | 9139 |
| c-Myc | - | 1:1000, BSA | 57-65 | 18583 |
| Vimentin | 1:200 | - | 57 | 5741 |
| βIII-tubulin | 1:400 | - | 55 | 5568 |
| PTEN | - | 1:1000, BSA | 54 | 9559 |
| GFAP | 1:200 | - | 50 | C9205  (Sigma-Aldrich) |
| TSG101 | - | 1:1000. BSA | 49 | 77452  (Novus Biologicals) |
| β-actin | - | 1:2000, TBST | 42 | A2228  (Sigma-Aldrich) |
| Bcl-xL | - | 1:1000, milk | 30 | 2764 |
| CD9 | - | 1:1000, BSA | 22, 24, 35 | 13403 |
| CD81 | - | 1:1000, BSA | 8, 25 | 65805  (Novus Biologicals) |
| CD63 | - | 1:1000, BSA | 6, 25 | 42225  (Novus Biologicals) |
| RhoA | - | 1:1000, BSA | 21 | 2117 |
| Bax | - | 1:1000, BSA | 20 | 14796 |
| Cleaved Caspase-3 | - | 1:1000, BSA | 17, 19 | 9664 |
| Iba1 | 1:200 | - | 17 | 17198 |
| S100β | 1:400 | 1:1000, BSA | 10 | 90393 |
